# Genome-wide transcriptional profiling of peripheral blood leukocytes from cattle infected with *Mycobacterium bovis *reveals suppression of host immune genes

**DOI:** 10.1186/1471-2164-12-611

**Published:** 2011-12-19

**Authors:** Kate E Killick, John A Browne, Stephen DE Park, David A Magee, Irene Martin, Kieran G Meade, Stephen V Gordon, Eamonn Gormley, Cliona O'Farrelly, Karsten Hokamp, David E MacHugh

**Affiliations:** 1 UCD College of Agriculture, Food Science and Veterinary Medicine, University College Dublin, Belfield, Dublin 4, Ireland; 2 Animal Bioscience Centre, Teagasc, Grange, Dunsany, County Meath, Ireland; 3 UCD Conway Institute of Biomolecular and Biomedical Research, University College Dublin, Belfield, Dublin 4, Ireland; 4 Tuberculosis Diagnostics and Immunology Research Centre, UCD School of Veterinary Medicine, University College Dublin, Belfield, Dublin 4, Ireland; 5 Comparative Immunology Group, School of Biochemistry and Immunology, Trinity College, Dublin 2, Ireland; 6 Smurfit Institute of Genetics, Trinity College Dublin, Dublin 2, Ireland

## Abstract

**Background:**

*Mycobacterium bovis *is the causative agent of bovine tuberculosis (BTB), a pathological infection with significant economic impact. Recent studies have highlighted the role of functional genomics to better understand the molecular mechanisms governing the host immune response to *M. bovis *infection. Furthermore, these studies may enable the identification of novel transcriptional markers of BTB that can augment current diagnostic tests and surveillance programmes. In the present study, we have analysed the transcriptome of peripheral blood leukocytes (PBL) from eight *M. bovis*-infected and eight control non-infected age-matched and sex-matched Holstein-Friesian cattle using the Affymetrix^® ^GeneChip^® ^Bovine Genome Array with 24,072 gene probe sets representing more than 23,000 gene transcripts.

**Results:**

Control and infected animals had similar mean white blood cell counts. However, the mean number of lymphocytes was significantly increased in the infected group relative to the control group (*P *= 0.001), while the mean number of monocytes was significantly decreased in the BTB group (*P *= 0.002). Hierarchical clustering analysis using gene expression data from all 5,388 detectable mRNA transcripts unambiguously partitioned the animals according to their disease status. In total, 2,960 gene transcripts were differentially expressed (DE) between the infected and control animal groups (adjusted *P*-value threshold ≤ 0.05); with the number of gene transcripts showing decreased relative expression (1,563) exceeding those displaying increased relative expression (1,397). Systems analysis using the Ingenuity^® ^Systems Pathway Analysis (IPA) Knowledge Base revealed an over-representation of DE genes involved in the *immune response *functional category. More specifically, 64.5% of genes in the *affects immune response *subcategory displayed decreased relative expression levels in the infected animals compared to the control group.

**Conclusions:**

This study demonstrates that genome-wide transcriptional profiling of PBL can distinguish active *M. bovis*-infected animals from control non-infected animals. Furthermore, the results obtained support previous investigations demonstrating that mycobacterial infection is associated with host transcriptional suppression. These data support the use of transcriptomic technologies to enable the identification of robust, reliable transcriptional markers of active *M. bovis *infection.

## Background

Bovine tuberculosis (BTB) poses a serious threat to the health of domestic cattle herds worldwide. Infection is caused by the bacterium *Mycobacterium bovis*, an intracellular pathogen closely related to *Mycobacterium tuberculosis*-the causative agent of human tuberculosis. *M. bovis *infection is often slow and progressive with limited clinical symptoms. Although improved diagnostic tests and slaughter policies have done much to control and reduce the incidence of infection, BTB has remained recalcitrant to eradication in many countries where control programmes have been implemented [1-3].

Failure to detect and remove all infected animals from herds is partly due to limitations in the sensitivity of the current diagnostic tests, which often comprise an *in vivo *single intradermal comparative tuberculin test (SICTT) performed alone, or in combination with an *in vitro *enzyme-linked immunosorbent assay (ELISA)-based test for interferon gamma (IFN-γ)-an established biomarker of mycobacterial infection [4-6]. Diagnoses can be further confounded by exposure to environmental non-pathogenic mycobacterial antigens, which can generate false SICTT-positive signals in cattle [7]. Protection from natural *M. bovis *infection in cattle may be achieved through vaccination with *M. bovis *bacillus Calmette-Guérin (BCG); however, the level of protection attained is variable. In addition, current diagnostics cannot effectively differentiate between *M. bovis*-infected and BCG-vaccinated animals, thus compromising management strategies [8]. Consequently, there is a pressing need for novel *M. bovis *diagnostic methods with increased sensitivity and specificity.

The host immune response to mycobacterial infection is a complex process that involves interaction between the innate and adaptive immune systems. Upon initial exposure (generally via inhalation), bacilli are phagocytosed by host alveolar macrophages, which recognise mycobacteria using a diverse range of pathogen recognition receptors (PRRs), such as the Toll-like receptors (TLRs) and the nucleotide-binding oligomerisation domain (NOD)-like receptors (NLRs) [9-13]. Activation of macrophage PRR-mediated signalling pathways result in the release of endogenous cytokines, which initiate an adaptive immune response characterised by the secretion of proinflammatory cytokines, such as IFN-γ and tumour necrosis factor (TNF-α), by activated T cells [14]. In particular, IFN-γ activates infected macrophages and enables the formation of granulomas-collections of inflammatory cells comprising T cells, B cells, non-infected macrophages and neutrophils, which surround infected macrophages and act as barriers to contain and prevent dissemination of the infection [15]. In most cases, the host innate and adaptive immune systems successfully control mycobacterial growth within granulomas resulting in asymptomatic latent infection [16,17]. However, in some cases impairment of immune function can result in the development of active tuberculosis leading to disease progression [3,17-20].

Recently, functional genomic technologies have been used to investigate the molecular mechanisms and cellular pathways underlying the host immune response to mycobacterial infection [[21,22], for reviews see [23,24]]. Furthermore, results from these studies have the potential to identify molecules that are critical for host/pathogen survival during infection, and which may serve as robust, reliable transcriptional markers of mycobacterial infection [22].

Previously, we investigated the transcriptional profiles of peripheral blood mononuclear cells (PBMC) from *M. bovis*-infected and non-infected control animals using the immuno-specific BOTL-5 microarray (containing 1,391 gene probe sets; Gene Expression Omnibus [GEO] accession: GPL5751) and showed that suppression of innate immune genes was associated with BTB [25]. In the current study, we extend this earlier work by investigating the transcriptional profile of peripheral blood leukocytes (PBL) isolated from eight *M. bovis*-infected and eight non-infected control animals using the genome-wide high-density Affymetrix^® ^GeneChip^® ^Bovine Genome Array. These 16 animals were sampled specifically for the present study and have not been used for any previous research work. The Affymetrix^® ^GeneChip^® ^Bovine Genome Array contains 24,072 gene probe sets representing more than 23,000 gene transcripts http://www.affymetrix.com. In addition, we have adopted a systems biology approach using the Ingenuity^® ^Systems Pathway Analysis (IPA) Knowledge Base http://www.ingenuity.com for analysis of both over-represented cellular functions and known molecular canonical pathways from the resulting gene expression data.

The results presented in the current study contribute a novel layer of information regarding the gene expression profile of PBL from *M. bovis*-infected animals and highlight the value of high-throughput genomic technologies in understanding the host immune response to BTB. Furthermore, these results may facilitate the development of novel diagnostics for the detection of *M. bovis *infection in domestic herds.

## Methods

### Experimental animals

Sixteen age-matched female Holstein-Friesian animals from cattle herds that had not been analysed previously were used for this study. Eight infected individuals were selected from a panel of naturally *M. bovis*-infected animals maintained for on-going disease surveillance at the Irish Department of Agriculture, Fisheries and Food, Backweston Laboratory Campus (Celbridge, Co. Kildare, Ireland). These animals had a positive single intradermal comparative tuberculin test (SICTT) result where the skin-fold thickness response to purified protein derivative (PPD)-bovine exceeded that of PPD-avian by at least 12 mm. All of these animals were also positive for the whole blood IFN-γ-based BoviGAM^® ^assay [Prionics AG, Switzerland] (data not shown). In addition, these cattle were confirmed for BTB following detailed post-mortem pathological examination and/or culture. Briefly, bronchial, mediastinal, submandibular, retropharyngeal, mesenteric and hepatic lymph nodes and lungs were examined macroscopically for tuberculosis lesions. Suspected lesions were cultured on Stonebrinks and Lowenstein-Jensen media at 37°C for eight weeks to detect *M. bovis *[26]. Non-infected control animals were selected from a herd with no recent history of *M. bovis *infection. The control animals were shown to be negative for both the SICTT and IFN-γ tests (data not shown). All animal procedures detailed in this study were carried out according to the provisions of the Cruelty to Animals Act (licenses issued by the Department of Health and Children) and ethics approval for the study was obtained from the UCD Animal Ethics Committee.

### Blood collection

Two 8 ml vacutainers^® ^(Becton-Dickinson Ltd., Dublin, Ireland) of heparinised blood were collected from each animal, approximately 12 months after positive SICTT testing. One vacutainer^® ^was retained for haematological analysis using a Cell-Dyn 3500 haematology analyser (Abbott Laboratories Ireland Ltd., Dublin, Ireland); all haematological analysis was performed using 1 ml of blood. The other vacutainer^® ^was used for RNA isolation from peripheral blood leukocytes (PBL); the whole white blood cell fraction consisting of T and B lymphocytes, NK cells, monocytes, neutrophils, basophils and eosinophils. The count data from the leukocyte cell populations of infected and non-infected animals were assessed using the two-sample, two-tailed Student's *t*-test, following Kolmogorov-Smirnov tests of normality and Levene's *F*-test for equality of variance using the Minitab statistical package version 16 (Minitab Ltd., Coventry, UK).

### RNA extraction and microarray analysis

All RNA extractions were performed within two hours of blood collection. Briefly, 7.5 ml of whole heparinised blood was mixed with 42.5 ml of erythrocyte blood lysis buffer (10 mM KHCO_3_, 150 mM NH_4_Cl, 1 mM EDTA pH 8.0), and incubated for 5 min at room temperature with gentle agitation. Following centrifugation (750 g for 10 min) the pelleted cells were washed once with 1× phosphate buffered saline (Invitrogen Ltd., Paisley, UK). The cell pellet was then fully resuspended in 2 ml Trizol^® ^reagent (Invitrogen Ltd., Paisley, UK) and RNA was extracted as per the manufacturer's instructions. The RNA was further purified using an RNeasy^® ^kit with on-column DNase treatment (Qiagen Ltd., Crawley, UK) according to the manufacturer's instructions. RNA quantity and quality was assessed using both the NanoDrop™ 1000 spectrophotometer (Thermo Fisher Scientific, Waltham, MA, USA) and the Agilent 2100 Bioanalyzer using an RNA 6000 Nano LabChip kit (Agilent Technologies, Cork, Ireland). All samples displayed a 260/280 ratio greater than 1.8 and RNA integrity numbers (RIN) greater than 8.0.

cDNA labelling, hybridisation and scanning for the microarray experiments were performed by Almac Diagnostics (Craigavon, Co. Armagh, Northern Ireland) using a one-cycle amplification/labelling protocol on the Affymetrix^® ^GeneChip^® ^Bovine Genome Array (Affymetrix UK Ltd., High Wycombe, UK).

### Statistical analysis of microarray data

Affymetrix^® ^GeneChip^® ^Bovine Genome Array data were analysed using Bioconductor [[27]; http://www.bioconductor.org] contained within the R statistical package http://www.r-project.org. Normalisation of raw data was performed using the Factor Analysis for Robust Microarray Summarization (FARMS) algorithm. The FARMS algorithm uses only perfect match (PM) probes and a quantile normalization procedure, providing both *P*-values and signal intensities [28]. In addition, gene expression profiles for each animal were clustered using the Hierarchical Ordered Partitioning and Collapsing Hybrid (HOPACH) clustering algorithm in Bioconductor with Euclidean distance as the distance metric [29].

Normalised data were then further subjected to filtering for informative probes sets using the R package I/NI-calls [30]. This defines a probe set as being informative when many of its probes reflect the same change in mRNA concentration across arrays. Differentially expressed genes were extracted using the Linear Models for Microarray Data (LIMMA) package http://bioconductor.org/packages/release/bioc/html/limma.html contained within the R statistical package. Genes displaying differential expression patterns between control and infected groups were annotated using the Affymetrix^® ^bovine gene annotation http://www.affymetrix.com. The Benjamini-Hochberg multiple-testing correction method [31] was applied to all differentially expressed genes to minimise the false discovery rate (FDR) and adjusted *P*-values for differentially expressed (DE) genes were calculated. For genes represented by multiple probe sets the mean expression value is reported. Bootstrapping for the hierarchical clustering analysis was performed using a custom Perl script (see Additional file 1).

### Systems biology analyses

Ingenuity^® ^Systems Pathway Analysis (IPA, Ingenuity Systems, Redwood City, CA, USA; http://www.ingenuity.com) was used to identify canonical pathways and functional processes of biological importance within the list of DE genes. The Ingenuity^® ^Knowledge Base contains the largest database of manually-curated and experimentally-validated physical, transcriptional and enzymatic molecular interactions. Furthermore, each interaction in the Ingenuity^® ^Knowledge Base is supported by previously published information.

For the IPA analyses, the Affymetrix^® ^GeneChip^® ^Bovine Genome Array was used as a reference gene set. All DE genes with an adjusted *P *value ≤ 0.05 were included. For duplicate probe IDs, the average log_2 _expression fold change was used. Only DE genes mapping to molecules in the Ingenuity^® ^Knowledge Base were used for systems analysis. Functional analysis of genes was performed using IPA to characterise the biological functions of the DE genes between the BTB and control groups. For this, IPA performed an over-representation analysis that categorises the DE genes within the uploaded list into functional groups using the Ingenuity^® ^Knowledge Base. Each category in IPA is ranked based on the number of DE genes falling into each functional group. Right-tailed Fisher's exact tests were used to calculate a *P*-value for each of the biological function assigned to list of DE genes.

IPA contains a large library of known canonical pathways that were overlaid with the DE genes to identify major biological pathways associated with *M. bovis *infection in PBL. The significance of the association between DE genes and the canonical pathway was assessed using two methods: (1) a ratio of the number of molecules from the DE gene data set that map to the pathway, compared to the total number of molecules that map to the canonical pathway based on the reference gene list; and (2) a Fisher's exact test that generates a *P*-value for the assignment of the DE genes to a particular canonical pathway compared to the reference gene list. Canonical pathways were then overlaid with the expression values of the DE genes.

### Real time quantitative reverse transcription PCR (qRT-PCR) validation of microarray results

cDNA was prepared using 500 ng of total RNA from each sample from the microarray study using the High Capacity cDNA Reverse Transcription Kit (Applied Biosystems, Warrington, UK) in a 20 μl reaction using random primers according to the manufacturer's instructions. cDNA was diluted 1:50 and stored at -20°C prior to performing real time quantitative reverse transcription PCR (qRT-PCR).

Real time qRT-PCR reactions were performed using Fast SYBR^® ^Green Master mix (Applied Biosystems, Warrington, UK) on a 7500 Fast Real-Time PCR System apparatus (Applied Biosystems, Warrington, UK). Each reaction (20 μl) contained 5 μl of the diluted cDNA (equivalent to 2.5 ng of total RNA) and 300 nM final concentration each of forward and reverse primer. Additional file 2, Table S1 provides a complete list of primer sequences and the target accession numbers for each real time qRT-PCR amplicon analysed. Real time qRT-PCR primers were designed using the Primer3 software [32,33] and where possible intron-spanning primers were selected (see Additional file 2, Table S1). Negative real time qRT-PCR controls and a six-point, four-fold dilution series from pooled cDNA from all animals were included on every real time qRT-PCR plate and individual PCR efficiencies were determined from the standard curves using the qbase^PLUS ^software package [[34]; Biogazelle NV, Zwijnaarde, Belgium].

The PCR thermal cycling program consisted of one cycle at 50°C for 2 min, one cycle at 95°C for 10 min, followed by 40 cycles at 95°C for 15 s and 60°C for 1 min. A dissociation step was included to confirm amplification specificity and real time qRT-PCR products were analysed on a 2% agarose gel to confirm the presence of a single discrete amplicon of the correct size. All real time qRT-PCR data was analysed using the qbase^PLUS ^software package with efficiency correction and normalization was performed using two reference genes: the 60S ribosomal protein L19 gene (*RPL19*) and peptidylprolyl isomerase A (cyclophilin A) gene (*PPIA*). The two reference genes were selected using the geNorm algorithm in the qbase^PLUS ^package from a panel of eight genes tested (geNorm M > 0.15).

The qbase^PLUS ^package generated a calibrated normalized relative quantity (CNRQ) of gene expression for each of the analysed samples. Log_2 _CNRQ values for both the control and the *M. bovis*-infected animals were used for statistical analysis for all genes. One sample Kolmogorov-Smirnov tests, performed using the SPSS^® ^version 18 software package (SPSS^® ^Inc., Chicago, IL, USA) were applied to the residuals of the log_2 _CNRQ values for each sample prior to statistical analysis to ensure the data conformed to a normal distribution—no significant departures from normality were observed for any of the genes analysed (*P *≥ 0.05). Two-tailed, two-sample Student *t*-tests were used to assess differences between infected and control groups based on log_2 _CNQR values using SPSS and the statistical package contained within Microsoft^® ^Excel 2010 (Microsoft Corp., Redmond, WA, USA). In addition, Levene's *F*-test was applied to the infected and control group log_2 _CNRQ values to assess equality of variance within the two groups using SPSS prior to performing the two-sample *t*-tests; equality of variance was observed for each sample group.

Geometric mean fold-changes in gene expression for the BTB group were calculated by dividing the geometric mean CNRQ value for the *M. bovis*-infected group by the geometric mean CNRQ value for the control group. The negative reciprocal in fold-change is reported where decreased gene expression was observed in the BTB group relative to the control group.

## Results

### Analysis of leukocyte populations in control and *M. bovis*-infected blood samples

The infected animals used in this study were chosen on the basis of their large responses to the comparative tuberculin skin test. The IFN-γ levels measured in whole blood of the infected animals were at least 25-fold greater than in the healthy control cattle (*P *< 0.001, data not shown), demonstrating that the infected animals were generating strong cell-mediated immune responses. At post-mortem, each of the infected animals displayed gross tuberculosis lesions in the lungs and thoracic lymph nodes and were classified as being in the advanced stage of clinical disease.

**Figure 1:**
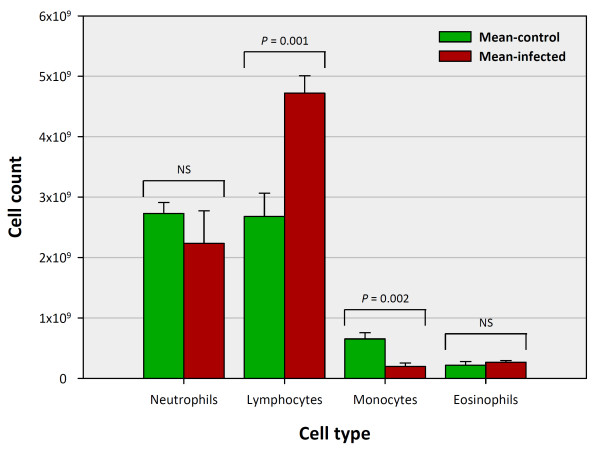
**Mean leukocyte cell population subset counts in control and *M. bovis*-infected animals (*n *= 8 each group)**. Error bars represent the standard error for each mean cell count estimate.

To assess potential changes in leukocyte composition between control and *M. bovis*- infected samples, whole blood samples were subjected to haematological analysis (Figure 1). No significant difference was observed in the total white blood cell (WBC) count between control and *M. bovis*-infected animals (*P *= 0.18). However, significant increases in the mean number of lymphocytes (*P *= 0.001) and significant decreases in the mean number of monocytes (*P *= 0.002) were observed in *M. bovis*-infected animals relative to the control animals. No significant differences in the mean number of eosinophils and neutrophils were observed between the two sample groups (*P *= 0.51 and *P *= 0.37, respectively).

### Summary of differentially expressed genes between control and *M. bovis*-infected animals identified from Affymetrix^® ^GeneChip^® ^analysis

**Figure 2:**
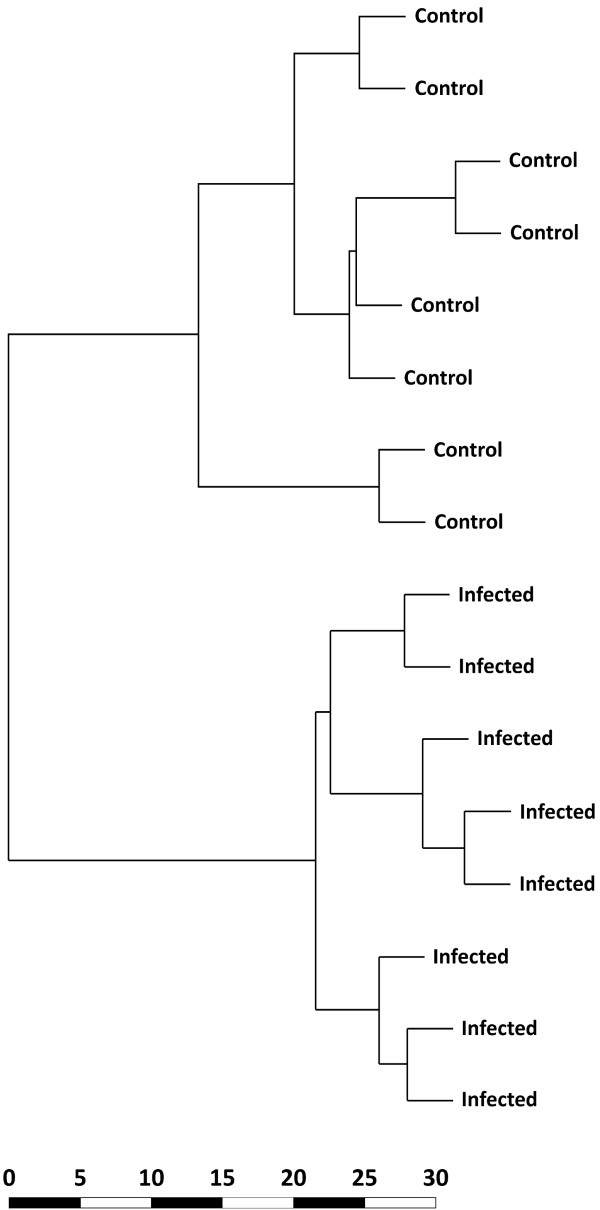
**Hierarchical clustering dendrogram generated using the complete gene expression profiles for the *M. bovis*-infected and control animals**. The dendrogram was generated from the complete gene expression dataset for each animal using the Euclidean distance metric. The division between the BTB and control animal groups was supported by a bootstrap value of 100%.

The expression data generated for the current study are MIAME-compliant [35] and were deposited in the NCBI Gene Expression Omnibus (GEO) repository [36] with experiment series accession GSE33359. All array transcripts that passed informative probe filtering were used for cluster analysis to examine the grouping of samples based on infection status (Figure 2). In total, 5,388 transcripts passed the filtering process and were used for the cluster analysis, which showed a clear partitioning of samples based on their disease status, indicating a distinct difference in expression profile between the two sample groups. The division of the two sample groups was supported by a 100% bootstrap value after 1,000 permutations.

Genome-wide transcriptional profiles generated from the PBL of eight *M. bovis*-infected and eight control animals were compared to assess differential gene expression between the two sample groups. The microarray analysis revealed a total of 2,960 transcripts, representing 2,757 unique genes, as differentially expressed between the *M. bovis*-infected and non-infected control animals (adjusted *P *value ≤ 0.05). In summary, 1,397 transcripts (representing 1,281 unique genes) displayed increased expression and 1,563 transcripts (representing 1,476 unique genes) displayed decreased expression in the *M. bovis*-infected group relative to the control group.

Among the DE genes with immune-related functions were genes encoding proinflammatory cytokines and other mediators of the host immune response also displayed increased relative expression in the BTB group. These included the cluster of differentiation 83 antigen-encoding gene (*CD83*) [+4.92-fold]; the chemokine (C-C motif) ligand 2 gene (*CCL2*) [+2.85-fold]; the chemokine (C-X-C motif) ligand 5 gene (*CXCL5*) [+3.87-fold]; the cytotoxic T-lymphocyte-associated protein 4 gene (*CTLA4*) [+3.20-fold]; the chemokine (C-X-C motif) receptor 4 gene (*CXCR4*) [+2.64-fold]; the interleukin 8 gene (*IL8*) [+2.15-fold] and the interleukin 1 alpha gene (*IL1A*) [+1.89-fold]. The genes encoding the TNF-α (*TNF*) and IFN-γ (*IFNG*) cytokines, two recognised biomarkers of *M. bovis *infection, were not differentially expressed between the two groups.

Reduced relative expression of host immune-related genes was also observed in the *M. bovis*-infected group relative to the control group. Among these were the antimicrobial beta-defensin 10 gene (*DEFB10*) [-3.38-fold]; the triggering receptor expressed on myeloid cells 1 gene (*TREM1*) [-1.70-fold]; and the TYRO protein tyrosine kinase binding protein gene (*TYROBP*) [-1.38-fold]. Other immune genes displaying reduced relative gene expression in the BTB group included those encoding proinflammatory cytokines such as the interleukin 15 gene (*IL15*) [-1.49-fold]; the interleukin 16 gene (*IL16*) [-1.44-fold]; and the interleukin 18 gene (*IL18*) [-1.72-fold].

Notably, several genes involved in TLR-mediated signalling displayed reduced relative expression in the BTB group such as the Toll-like receptor 4 gene (*TLR4*) [+2.41-fold]; the Toll-like receptor 2 gene (*TLR2*) [-1.45-fold]; the TLR adaptor protein myeloid differentiation primary response gene (88) [*MYD88*] (-1.31-fold); the interleukin-1 receptor-associated protein kinase 4 gene (*IRAK4*) [-1.11-fold] and the mitogen-activated protein kinase 13 and 14 genes (*MAPK13 *[-1.93-fold] and *MAPK14 *[-1.15-fold]). The gene encoding the intracellular TLR3 protein (*TLR3*) also displayed reduced relative expression in the BTB animals (-1.55-fold).

### Real time quantitative reverse transcription PCR (qRT-PCR) analysis and validation of Affymetrix^® ^GeneChip^® ^results

A panel of 23 immune-related genes, including members of the interferon signalling pathway, which was recently shown to be the most significantly over-represented pathway in human patients with active TB [37], were selected for real time qRT-PCR analysis. These were *CASP1, CD83, CTLA4, DEFB10, IFNB, IFNAR1, IFNG, IFNGR1, IFNGR2, IL1A, IL8, IL15, JAK1, KIR3DS1, MYD88, PTPN2, STAT1, STAT2, TLR3, TLR4, TREM1, TYK2 *and *TYROBP*. The immune-related function of each gene and the results from these analyses are detailed in Table 1.

**Table 1:** Gene expression fold-changes between *M.bovis*-infected (*n *= 8) and control (*n *= 8) based on microarray and real time qRT-PCR analyses

Gene symbol	Gene name	Gene description	Mean *M.bovis*-infected *vs *control group expression fold-change (microarray)	Mean *M.bovis*-infected *vs *control group expression fold-change (real time qRT-PCR)	Real time qRT-PCR *P*-value
*CASP1*	Caspase 1 gene	A member of the cysteine-aspartic acid protease. Plays a role in the cell apoptosis	-1.45	-1.98	0.001

*CD83*	Cluster of differentiation 83 gene	A cell surface protein found in antigen presenting cells. Believed to play a role in antigen presentation or the cellular interactions that follow lymphocyte activation	+4.92	+5.88	< 0.001

*CTLA4*	Cytotoxic T-lymphocyte-associated protein 4 gene	A member of the immunoglobulin superfamily and encodes a protein which transmits an inhibitory signal to T cells	+3.20	+3.46	0.001

*DEFB10*	Beta-defensin 10 gene	Host defence response to bacterial infection; has antimicrobial activity	-3.38	-12.21	0.018

*IFNB1*	interferon, beta 1, fibroblast	Cytokine activity	Not DE	Not DE	> 0.05

*IFNAR1*	Interferon (alpha, beta and omega) receptor 1 gene	A type I membrane protein that forms one of the two chains of a receptor for interferons alpha and beta	Not DE	-2.20	< 0.001

*IFNG*	Interferon gamma gene	A soluble cytokine with antiviral, immunoregulatory and anti-tumor properties. A potent activator of macrophages	Not DE	Not DE	> 0.05

*IFNGR1*	Interferon gamma receptor 1 gene	Forms a heterodimer with interferon gamma receptor 2. Involved in binding of interferon gamma	Not DE	+2.23	< 0.001

*IFNGR2*	Interferon gamma receptor 2 gene	Forms a heterodimer with interferon gamma receptor 1. Involved in binding of interferon gamma	-1.32	Not DE	> 0.05

*IL1A*	Interleukin 1, alpha gene	A member of the interleukin 1 cytokine family produced by macrophages. Involved in various immune responses, inflammatory processes, and haematopoiesis	+1.89	+2.47	0.009

*IL8*	Interleukin 8 gene	A chemokine that mediates the inflammatory response	+2.15	+4.01	< 0.002

*IL15*	Interleukin 15 gene	A cytokine that regulates T and natural killer cell activation and proliferation	-1.49	-2.03	< 0.001

*JAK1*	Janus kinase 1 gene	A widely expressed membrane-associated phosphoprotein involved in the interferon-alpha/beta and -gamma signal transduction pathways	Not DE	Not DE	> 0.05

*KIR3DS1*	Killer cell immunoglobulin-like receptor, three domains, short cytoplasmic tail, 1	A transmembrane glycoprotein expressed by natural killer cells and some T cells. Involved in regulation of the immune response	-1.44	-2.63	0.005

*MYD88*	myeloid differentiation primary response gene (88)	A cytosolic adapter protein that functions as a signal transducer in the interleukin-1 and Toll-like receptor signalling pathways	-1.31	-2.55	< 0.001

*PTPN2*	protein tyrosine phosphatase, non-receptor type 2 gene	A member of the protein tyrosine phosphatase (PTP) family that is involved in a variety of cellular processes including cell growth, and differentiation	Not DE	-1.43	< 0.001

*STAT1*	signal transducer and activator of transcription 1, 91kDa gene	A transcriptional activator protein activated in response to cytokines and growth factors	+1.28	-1.27	< 0.001

*STAT2*	signal transducer and activator of transcription 2, 113kDa gene	A transcriptional activator protein activated in response to cytokines and growth factors	-1.23	-1.86	< 0.001

*TLR3*	Toll-like receptor 3 gene	An intracellular pathogen recognition receptor (PRR) that largely recognises viral pathogen-associated molecular patterns (PAMPs). Mediates the production of cytokines necessary for the development of effective immunity	-1.55	-1.63	0.001

*TLR4*	Toll-like receptor 4 gene	A cell-surface PRR that recognises PAMPs expressed by infectious agents. Mediates the production of cytokines necessary for the development of effective immunity	+2.41	Not DE	> 0.05

*TREM1*	Triggering receptor expressed on myeloid cells 1 gene	A receptor expressed on myeloid cells upon microbial infection. Amplifies neutrophil and monocyte-mediated inflammatory responses stimulating release of pro-inflammatory chemokines and cytokines	-1.70	-2.81	0.002

*TYK2*	Tyrosine kinase 2 gene	A member of the tyrosine kinase. A component of both interferon signalling pathways	Not represented on array	-1.60	< 0.001

*TYROBP*	TYRO protein tyrosine kinase binding protein gene	A transmembrane protein involved in cell signalling	-1.38	-1.98	< 0.001

Gene descriptions taken from GeneCards version 3 [82] and the NCBI Entrez Gene database [83]. For the microarray data, genes with fold-change differences in expression were significant after adjustment for multiple testing using the Benjamini-Hochberg method [31] (adjusted *P *≤ 0.05). 'Not DE' indicates a gene was not differentially expressed between the two sample groups.

Thirteen of the 23 genes analysed using real time qRT-PCR (*CASP1, DEFB10, IFNAR1, IL15, KIR3DS1, MYD88, PTPN2, STAT1, STAT2, TLR3, TREM1, TYK2 *and *TYROBP*) showed significant decreased expression (*P *≤ 0.05) and five genes (*CD83, CTLA4, IFNGR1, IL1A *and *IL8*) displayed significant increased expression (*P *≤ 0.05) in the *M.bovis*-infected group relative to the control animals. No statistically significant differences in expression (*P *> 0.05) were observed between the two sample groups for the remaining five genes assayed (*IFNB1, IFNG, IFNGR2, JAK1*and *TLR4*).

Twenty-two of the 23 genes analysed by real time qRT-PCR were represented on the microarray; only *TYK2 *was not represented. Gene expression profiles for 16/22 (73%) of the genes analysed with real time qRT-PCR were concordant with the results from the microarray analysis. Two genes (*IFNGR2 *and *TLR4*) displayed significant expression differences between the two sample groups based on the microarray results, but were not significantly different based on real time qRT-PCR data analysis. Three genes (*IFNAR1, IFNGR1 *and *PTPN2*) displayed significant differences between the two groups based on real time qRT-PCR results; however, these genes were not differentially expressed according to the microarray results. Only one gene (*STAT1*) displayed directionally discordant gene expression profiles between the two methods. In the *M. bovis*-infected group, *STAT1 *showed significant reduced relative expression (-1.27-fold) based on real time qRT-PCR results but displayed significant increased relative expression (+1.28-fold) according to the microarray results. The observed discrepancies between the microarray and real time qRT-PCR data may reflect differences in the sensitivity of the two analytical methods used and/or differences in the mRNA transcripts targeted by the probes (microarray) and primer pairs (real time qRT-PCR) used for the two forms of gene expression analysis.

### Analysis of differential gene expression using Ingenuity^® ^Systems Pathway Analysis (IPA)

**Figure 3:**
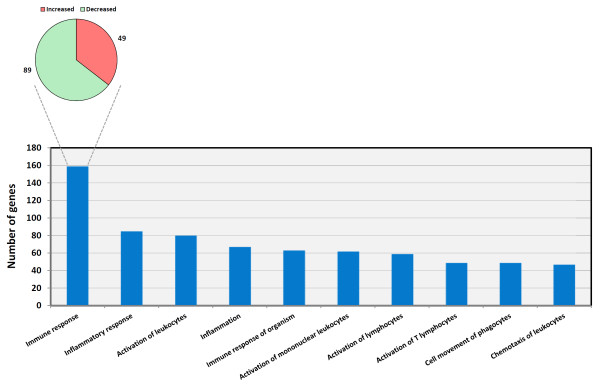
**Sub-categories of the top ranking IPA-identified *inflammatory response *gene ontology (GO) category**. The numbers of genes displaying increased and decreased relative expression for *affects immune response*, the top ranking subcategory, are shown. The number of differentially expressed genes within each functional subcategory is indicated.

The total number of DE genes that could be mapped to molecules in the Ingenuity^® ^Knowledge Base was 1,869 from a total of 2,960 DE transcripts. IPA was used to categorise these 1,869 DE genes based on their functional annotation and to assess if a functional gene category contained an over-representation of genes relative to the microarray reference gene list. This analysis showed that the top functional category observed for the DE genes used for IPA was the *inflammatory response*, which contained 241 genes with *P*-values ranging from 1.66 × 10^-11 ^to 1.64 × 10^-2 ^(Table 2). The *inflammatory response *category was further sub-divided with the *affects immune response *subcategory containing the most molecules (138 DE genes) [Figure 3]. Further inspection of the individual genes within this subcategory revealed that there was an over-representation of genes displaying reduced relative expression (89 genes) compared to DE genes showing increased relative expression in the BTB animals (49 genes). This observation was in contrast to the other top functional categories where similar numbers of genes showing increased and decreased relative expression were reported.

**Table 2:** Gene ontology (GO) categories identified using IPA

Gene ontology category	*P*-value range	Number of genes
Inflammatory response	1.66 × 10^-11 ^- 1.64 × 10^-2^	241

Cellular development	8.95 × 10^-10 ^- 1.57 × 10^-2^	308

Cellular growth and proliferation	1.65 × 10^-9 ^- 1.57 × 10^-2^	333

Haematological system development and function	1.65 × 10^-9 ^- 1.64 × 10^-2^	279

Haematopoiesis	2.60 × 10^-8 ^- 1.57 × 10^-2^	173

Tissue morphology	1.78 × 10^-7 ^- 1.19 × 10^-2^	114

Cellular function and maintenance	5.44 × 10^-7 ^- 1.65 ×10^-2^	138

Cell death	9.57 × 10^-7 ^- 1.60 × 10^-2^	419

Cell-mediated immune response	1.06 × 10^-6 ^- 1.25 × 10^-2^	109

Connective tissue disorders	1.72 × 10^-6 ^- 5.94 × 10^-3^	234

Immunological disease	1.72 × 10^-6 ^- 1.57 × 10^-2^	344

Inflammatory disease	1.72 × 10^-6 ^- 1.28 × 10^-2^	364

Skeletal and muscular disorders	1.72 × 10^-6 ^- 1.22 × 10^-2^	235

Infectious disease	1.82 × 10^-6 ^- 1.17 × 10^-2^	223

Cell-to-cell signalling and interaction	8.33 × 10^-6 ^- 1.64 × 10^-2^	210

Dermatological diseases and conditions	1.04 × 10^-5 ^- 1.57 × 10^-2^	107

Free radical scavenging	1.23 × 10^-5 ^- 1.23 × 10^-5^	14

Molecular transport	1.23 × 10^-5 ^- 7.43 × 10^-3^	86

Cell signalling	2.40 × 10^-5 ^- 7.21 × 10^-5^	133

Respiratory disease	2.86 × 10^-5 ^- 1.24 × 10^-2^	109

Cellular compromise	3.09 × 10^-5 ^- 1.57 × 10^-2^	41

Genetic disorder	3.39 × 10^-5 ^- 1.64 × 10^-2^	104

Immune cell trafficking	3.51 × 10^-5 ^- 1.64 × 10^-2^	163

Humoral immune response	5.08 × 10^-5 ^- 1.19 × 10^-2^	78

Organismal injury and abnormalities	9.51 × 10^-5 ^- 1.05 × 10^-2^	81

Tissue development	1.19 × 10^-4 ^- 1.05 × 10^-2^	108

Haematological disease	1.78 × 10^-4 ^- 1.35 × 10^-2^	162

Gastrointestinal disease	1.88 × 10^-4 ^- 5.51 × 10^-3^	42

Hepatic system disease	1.88 × 10^-4 ^- 1.64 × 10^-2^	89

Lymphoid tissue structure and development	1.94 × 10^-4 ^- 5.94 × 10^-3^	5

Antigen presentation	3.42 × 10^-4 ^-1.64 × 10^-2^	83

Cellular movement	3.58 × 10^-4 ^- 1.63 × 10^-2^	157

Vitamin and mineral metabolism	3.59 × 10^-4 ^- 7.21 × 10^-3^	74

Antimicrobial response	3.92 × 10^-4 ^- 1.28 × 10^-2^	32

Hypersensitivity response	5.86 × 10^-4 ^- 8.60 × 10^-3^	31

Infection mechanism	9.89 × 10^-4 ^- 1.57 × 10^-2^	39

Gene expression	1.07 × 10^-3 ^- 1.57 × 10^-2^	254

Hair and skin development and function	1.23 × 10^-3 ^- 1.57 × 10^-2^	22

Post-translational modification	2.27 × 10^-3 ^- 9.78 × 10^-3^	84

Cancer	2.51 × 10^-3 ^- 1.57 × 10^-2^	385

Endocrine system development and function	2.65 × 10^-3 ^- 2.65 × 10^-3^	21

Carbohydrate metabolism	3.77 × 10^-3 ^- 3.77 × 10^-3^	6

Cell morphology	3.77 × 10^-3 ^- 1.28 × 10^-2^	23

Lipid metabolism	3.77 × 10^-3 ^- 7.43 × 10^-3^	17

Neurological disease	3.77 × 10^-3 ^- 1.47 × 10^-2^	49

Small molecule biochemistry	3.77 × 10^-3 ^- 7.43 × 10^-3^	23

Metabolic disease	3.91 × 10^-3 ^- 3.91 × 10^-3^	7

Embryonic development	4.59 × 10^-3 ^- 1.57 × 10^-2^	21

Nervous system development and function	4.59 × 10^-5 ^- 1.18 × 10^-2^	8

Reproductive system disease	5.84 × 10^-3 ^- 7.54 × 10^-3^	79

Cell cycle	5.94 × 10^-3 ^- 1.18 × 10^-2^	20

Endocrine system disorders	5.94 × 10^-3 ^- 5.94 × 10^-3^	3

Opthalmic disease	5.94 × 10^-3 ^- 6.60 × 10^-3^	7

RNA post-transcriptional modification	6.04 × 10^-3 ^- 6.04 × 10^-3^	13

Cardiovascular system development and function	6.68 × 10^-3 ^- 1.30 ×10^-2^	19

Cell assembly and modification	6.68 × 10^-3 ^- 1.28 × 10^-2^	14

Organ development	6.68 × 10^-3 ^- 1.19 × 10^-2^	6

Renal and urological system development and function	1.18 × 10^-2 ^- 1.30 × 10^-2^	11

Reproductive system development and function	1.18 × 10^-2 ^- 1.18 × 10^-2^	4

The top ranking GO categories identified by IPA are listed according to *P*-values. *P*-value ranges are based on subcategory *P*-values within each parent term.

**Figure 4:**
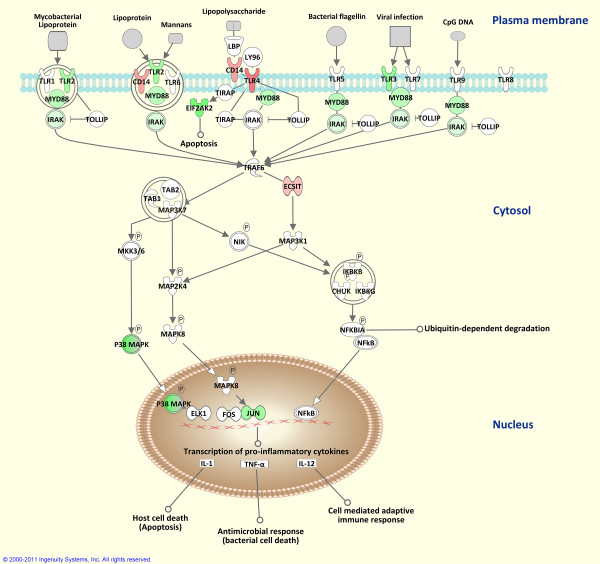
**Differential gene expression in the TLR signalling pathway**. Genes within the TLR signalling pathway showing differential expression are highlighted in colour. Colour intensity indicates the degree of increased (red) or decreased (green) relative expression in the BTB group compared to the control animal group. A white colour indicates genes that were not differentially expressed and entities coloured grey represent microbial PAMPs.

Canonical molecular pathways associated with *M. bovis*-infection were analysed using IPA. These canonical pathways were ranked according to *P*-value, which represents the significance of the association between a specific pathway and the genes in the input data set. The majority of the top ranking IPA-identified canonical pathways were involved in cell signalling and communication associated with host innate and adaptive immune responses. *Natural killer cell signalling *and *communication between innate and adaptive immune cells *were identified as the top ranking canonical pathways. In addition, *TREM1 signalling, dendritic cell maturation, JAK-STAT signalling, T cell signalling, IL6 signalling, chemokine signalling *and *TLR signalling *were among the top twenty IPA-identified canonical pathways (Table 3). Based on the well-documented role of TLR signalling in mycobacterial infection [13,38-42], this canonical pathway overlaid with gene expression results is shown in Figure 4.

**Table 3:** Top-ranking canonical pathways identified using IPA

Canonical Pathway Name	*P*-value	Ratio
Natural killer cell signalling	3.06 × 10^-4^	23/112 (0.205)

Communication between innate and adaptive immune cells	4.11 × 10^-4^	17/89 (0.191)

TREM1 signalling	2.20 × 10^-3^	15/69 (0.217)

Dendritic cell maturation	4.96 × 10^-3^	29/174 (0.167)

Cysteine metabolism	7.43 × 10^-3^	9/90 (0.100)

JAK/STAT signalling	8.22 × 10^-3^	15/64 (0.234)

NRF2-mediated oxidative stress response	8.98 × 10^-3^	35/183 (0.191)

T cell receptor signalling	1.08 × 10^-2^	20/107 (0.187)

IL6 signalling	1.52 × 10^-2^	20/93 (0.215)

CCR5 signalling in macrophages	1.84 × 10^-2^	16/92 (0.174)

Chemokine signalling	1.92 × 10^-2^	15/75 (0.200)

Calcium-induced T-lymphocyte apoptosis	2.07 × 10^-2^	12/66 (0.182)

IL-17 signalling	2.21 × 10^-2^	16/74 (0.216)

Prolactin signalling	2.21 × 10^-2^	16/75 (0.213)

Synaptic long term potentiation	2.22 × 10^-2^	19/113 (0.168)

Toll-like receptor signalling	2.63 × 10^-2^	11/54 (0.204)

FLT3 signalling in hematopoietic progenitor cells	2.77 × 10^-2^	15/74 (0.203)

Systemic lupus erythematosus signalling	2.81 × 10^-2^	20/151 (0.132)

Renin angiotensin signalling	3.01 × 10^-2^	21/120 (0.175)

Phospholipase C signalling	3.28 × 10^-2^	39/253 (0.154)

Oncostatin M signalling	3.36 × 10^-2^	9/35 (0.257)

Thyroid cancer signalling	3.36 × 10^-2^	9/42 (0.214)

B cell receptor signalling	3.72 × 10^-2^	25/154 (0.162)

Interferon signalling	3.97 × 10^-2^	6/30 (0.200)

Production of nitric oxide and reactive oxygen species in macrophages	4.13 × 10^-2^	28/185 (0.151)

NF-κB signalling	4.17 × 10^-2^	25/152 (0.164)

Notch signalling	4.29 × 10^-2^	8/43 (0.186)

IL10 signalling	4.36 × 10^-2^	13/70 (0.186)

P38 MAPK signalling	4.57 × 10^-2^	17/97 (0.175)

Role of NFAT in regulation of the immune response	4.70 × 10^-2^	29/196 (0.148)

Parkinson's signalling	5.00 × 10^-2^	5/17 (0.294)

The top ranking canonical pathways identified by IPA are listed according to *P*-values. The ratio indicates the number of differentially expressed genes involved in each canonical pathway divided by the total number of genes within each pathway as per the IPA Knowledge Base.

## Discussion

The implementation of surveillance and management programmes has done much to reduce the incidence and prevalence of BTB over the past number of decades; however, *M. bovis *infection remains an important livestock disease worldwide. This is due, in part, to well-documented limitations of the currently available diagnostics tests (such as the SICTT and IFN-γ tests) leading to a failure to detect all infected animals [43,44].

In recent years, research has shifted from a focus on protein-based diagnostics to functional genomics technologies that interrogate the host transcriptome in response to *M. bovis *infection. In particular, microarray technologies coupled with the rapid development of more sophisticated bovine genome resources has enabled high-resolution analyses of the genes and cellular pathways governing the host response to infection with *M. bovis *[23,25,45-47]. In the present study, we have compared the transcriptomes of PBL from non-infected control animals with actively-infected BTB animals using a high-density genome-wide bovine microarray platform.

Modulation of the host PBL transcriptome in response to *M. bovis *infection was evident from the large number of DE genes between the two experimental groups. Statistical analyses of the microarray data identified a total of 2,757 DE genes. Of these, 1,281 (46%) showed increased expression and 1,476 (54%) displayed decreased expression in the BTB group compared to the control animals. It is important to note, however, that the differences in cell subpopulations observed between the *M. bovis*-infected and control animals (Figure 1) may have contributed to the gene expression changes detected between the two experimental groups. Also, the haematology analyser results only provided a general description of the PBL cell subsets and do not provide information concerning T lymphocyte subsets in the infected and control animal groups. In addition, the cell subset results presented here differ from previous work performed by us [25], most likely due to the different cell sample types examined.

Analysis of the DE genes using IPA provided information regarding the immunobiology of active BTB. The highest ranking functional category identified using IPA was *inflammatory response *and the highest ranking subcategory within this category-*affects immune response*-revealed a marked bias in the number of genes displaying a decrease in relative expression (64.5%) compared to those showing an increase in relative expression (35.5%) in PBL from the BTB group.

Previous work by our research group demonstrated that suppression of host gene expression was associated with active *M. bovis *infection in cattle [25]. This earlier work involved the comparison of RNA isolated from PBMC of *M. bovis*-infected and control animals using an immuno-specific bovine cDNA microarray (BOTL-5). The results presented here, based on the analysis of PBL-derived RNA using a genome-wide microarray, lend further support to our previous study. Indeed, published investigations by other workers suggest that transcriptional suppression is a common feature of mycobacterial infection in mammals [48-50].

Further inspection of the *inflammatory response *functional category in IPA identified several genes that were previously reported to be differentially expressed in cattle and other mammalian species infected with mycobacterial pathogens [23,40,42,51,52]. For example, microarray analysis showed that *TLR2 *and *TLR4 *displayed contrasting expression patterns between PBL from the two groups: *TLR2 *showed decreased expression and *TLR4 *showed increased expression relative to the control animal group. We have previously observed decreased expression of *TLR2 *in PBMC from actively infected BTB cattle using the immuno-specific BOTL-5 cDNA microarray; however, contrary to the results of the present study, *TLR4 *also showed decreased expression with the BOTL-5 cDNA microarray in actively infected animals [25].

The gene expression results obtained by Meade and colleagues using PBMC from *M. bovis*-infected and control non-infected animals were also used to identify a panel of 15 genes predictive of disease status [25]. Four of these genes were found to be similarly differentially expressed in the current study: *UNC84B *(now *SUN2*), *GAN, SFPQ *and *NRP1*. Four other of the 15 genes identified previously (*TBK1, 28S *[now *RN28S1*], *GPR98 *and an anonymous BOTL clone [*BOTL0100013_F01*]) were not present on the Affymetrix^® ^GeneChip^® ^Bovine Genome Array. However, the seven remaining genes (*NCOR1, PPP2R5B, UCP2, ZDHHC19, NFKB1, NRM *and *FGFR1*) were not differentially expressed in the PBL samples from *M. bovis*-infected and control non-infected animals used for the present study. This discordance may be due to a number of factors, including: the blood cell sample types used (PBL versus PBMC); differences in sensitivity between the two types of microarray (the single-colour *in situ-*synthesised Affymetrix^® ^GeneChip^® ^versus a dual-colour spotted cDNA array [53,54]); and the requirement for more stringent control of the FDR with larger numbers of genes (24,072 probe sets versus 1,391 duplicate spot features).

The role of TLR molecules in the recognition of mycobacterial PAMPs is well established [11,38,39,41,42,55-57]. TLR2 and TLR4 activation signals are linked to the interleukin-1 receptor-associated protein kinases (IRAKs) through the adaptor molecule, myeloid differentiation primary response protein 88 (MYD88), which triggers a downstream protein signalling cascade involving tumour necrosis factor receptor-associated factor 6 (TRAF6) and mitogen-activated protein kinases (MAPKs) [58,59]. This cascade culminates in the expression of many NF-κB-inducible genes, including *CCL2, IL1B, IL12, IL18 *and *TNF*, causing natural killer (NK) and T cells to release IFN-γ and TNF-α, which ultimately results in granuloma formation [60].

In the present study, several TLR-mediated proinflammatory cytokines and signalling molecules were differentially expressed in the BTB group compared to the non-infected control animals. These included *CCL2 *(increased), *CXCR4 *(increased), *CXCL5 *(increased), *IL1A *(increased), *IL8 *(increased), *IL18 *(decreased), *IRAK4 *(decreased), *MAPK6 *(increased), *MAPK13 *(decreased), *MAPK14 *(decreased) and *MYD88 *(decreased). This was also supported by canonical pathway analysis using IPA, which identified TLR signalling as a molecular pathway affected by *M. bovis *infection.

These results suggest that genes encoding TLR-mediated signalling pathway molecules have a role in governing the host response to BTB and may also serve as targets for immuno-subversion by *M. bovis*. For example, genes encoding several innate immune receptors and chemokines (such as *TLR4, CD83, CCL2, CXCR4, CXCL5, IL1A *and *IL8*)—several of which participate in the initiation of a T cell response during infection [61-64]—showed increased relative expression in the BTB animal group. Transcriptional profiles suggesting initiation of a T cell response are supported by the comparative analysis of the PBL cell populations in the two animal groups; a significant increase in the mean number of lymphocytes and a significant decrease in the mean number of monocytes were observed in the BTB group relative to the control animals. This difference in the PBL cell composition may represent recruitment of host cytotoxic lymphocytes for the destruction of infected monocytes in the control of *M. bovis *infection [3,65].

It is important to note, however, that the observed decreased expression of host PRR genes (such as *TLR2*) and the genes encoding their associated adaptor and signalling pathway molecules (such as *MYD88, IRAK4, MAPK13 *and *MAPK14*) may indicate that the adaptive response in BTB animals is inferior due to the repression of these innate immune genes. Indeed, previous work has proposed that mycobacterial antigens, such as the early secreted antigenic target protein 6 (ESAT-6) protein, attenuates the host innate immune response by inhibiting MYD88-IRAK4 binding, thus causing suppression of NF-κB-induced transcription of upstream genes required for T cell response initiation [66]. These workers also demonstrated that activation of v-akt murine thymoma viral oncogene homolog kinases (AKTs) is necessary to prevent MYD88-IRAK4 complex formation. Notably, the *AKT2 *gene displayed increased relative expression (+1.22-fold) in the BTB animal group in the present study.

Repression of host innate immune genes that elicit an adaptive response to *M. bovis *infection is further supported by the analysis of genes belonging to the interferon signalling pathway, which has been shown to have a role in human tuberculosis [37,67-71]. IFN-γ is secreted by NK cells and CD4^+ ^T cells upon activation by IL-12 produced by infected macrophages. IFN-γ recruits additional macrophages to the site of infection while also providing the stimulus for activating microbicidal functions in infected macrophages [14,71-73]. IFN-γ also induces MHC class II gene expression in infected macrophages by signalling through its receptor (IFN-γ-receptor) [74-76]. This stimulates the JAK-STAT pathway, resulting in induction of transcriptional activators of MHC class genes, such as the MHC class II transactivator gene (*CIITA*). Mycobacterial antigen presentation via MHC class II molecules is critical for the recruitment of additional CD4^+ ^T cells and the formation and maintenance of granulomas [38].

The results from the current study support a role for interferon signalling pathways during *M. bovis *infection. The genes encoding interferon (alpha, beta and omega) receptor 2 (*IFNAR2*); interferon gamma receptor 2 (*IFNGR2*); interferon-induced protein with tetratricopeptide repeats 2 (*IFIT2*); interferon-induced protein with tetratricopeptide repeats 5 (*IFIT5*); interferon-induced transmembrane protein 3 (*IFITM3*); protein tyrosine phosphatase, non-receptor type 2 (*PTPN2*); and signal transducer and activator of transcription 2 (*STAT2*) displayed differential expression in the BTB animals based on the microarray and/or real time qRT-PCR analyses.

These findings suggest that, in addition to the targeting of TLR-mediated signalling pathways, *M. bovis *may also target genes involved in the IFN-signalling pathway, resulting in an attenuated T cell response that enables mycobacterial survival and disease progression. It is tempting to speculate that suppression of IFN-signalling in response to *M. bovis *infection may result in the impairment of the antigen presenting process required for adaptive immunity; however, further work is required to investigate this possibility. Notably, the gene encoding IFN-γ (*IFNG*) was not differentially expressed in the current study, despite the BTB animals having tested positive for increased IFN-γ based on the BoviGAM^® ^assay. However, it is important to note that unlike the blood samples used for the BoviGAM^® ^assay in the current work, the PBL fraction from which the RNA was derived in this study was not stimulated with protein purified derivative of tuberculin (PPD), which is required to elicit IFN-γ secretion [4]. In addition, contrary to previous results obtained by Meade and colleagues [25] we did not detect differential expression of the *TNF *gene between the *M. bovis*-infected and control animals examined here. The most likely explanation for this apparent discrepancy is the different cell sample types used for gene expression analyses (PBL versus PBMC).

IPA canonical pathway analysis identified a number of DE genes which, to our knowledge, have not previously been reported to be involved in the host response to tuberculosis in cattle or other mammalian species. These included *CTLA4 *and *TLR3*. *TLR3 *encodes an intracellular PRR involved in the recognition of viral-derived nucleic acids [77]. In the present study, reduced relative *TLR3 *expression in BTB animals (-1.55-fold) may suggest some hitherto unknown role for this PRR in intracellular mycobacterial infection. In support, we have observed significant differential expression of *TLR3 *in bovine monocyte-derived macrophages (MDM) stimulated *in vitro *with *M. bovis *when compared to non-stimulated control MDM (unpublished data). *CTLA4 *encodes an inhibitor of the T cell-mediated response [78,79] and this gene displayed increased relative expression (+3.20-fold, *P *≤ 0.05) in the *M. bovis *infected animals in the present study. The observed increased relative expression of *CTLA4 *may reflect a mechanism of immuno-modulation used by *M. bovis *to subvert a host T cell response.

Finally, hierarchical clustering analysis was performed here using a total of 5,388 genes that passed the informative probe filtering criteria. This analysis unambiguously differentiated animals on the basis of their disease status. This result suggests that genome-wide expression profiling of PBL from BTB animals can be used to enable the identification of suitable transcriptional markers for the detection of infected animals within herds and augment current surveillance strategies in countries where control programmes have been implemented [21,22]. However, further work using PBL samples from additional animals infected with *M. bovis *and other microbial pathogens will be required to identify and validate robust *M. bovis*-specific transcriptional signatures of infection.

## Conclusions

The results presented here support the hypothesis that repression of immune-related genes is an important feature of mycobacterial infections [25,48-50,80,81]. In particular, the gene expression results obtained suggest that *M. bovis *infection may target the innate immune cellular pathways necessary for the initiation of the appropriate T cell response. Notably, analysis of the cell populations present in the PBL from the BTB animals showed an increase in the number of lymphocytes relative to the control animals, suggesting that the actively-infected BTB animals do mount a T cell response. However, it is possible that the T cell response elicited by these animals is compromised, resulting in disease progression. Indeed, failure of the adaptive immune response to contain the mycobacterial infection is regarded as the primary cause of the development of active tuberculosis from a latent state of infection [74]. Finally, cluster analysis using all informative mRNA transcripts permitted a clear delineation between healthy and infected animals. These results demonstrate that functional genomics approaches based on transcriptional profiling can be used to supplement current protein-based diagnostics for BTB.

## Authors' contributions

KEK was responsible for statistical analyses of microarray data, systems biology analysis and manuscript preparation. JAB was responsible for coordinating microarray preparation, haematological analysis, real time qRT-PCR validation of results and manuscript editing. DAM performed statistical analyses and contributed to manuscript preparation and editing. SDEP and KH contributed to the bioinformatics, statistical analysis and systems biology components of the work. IM performed real time qRT-PCR validation of results. KGM and EG contributed to the experimental design and manuscript editing. SVG and COF contributed to the experimental design, provided valuable comments, discussion and contributed to manuscript preparation and editing. DEM was responsible for the experimental design, coordination of functional genomics data analyses and manuscript preparation and editing. All authors read and approved the final manuscript.

## Supplementary Material

Additional file 1 **Perl script used for bootstrapping of cluster analysis results in **Figure 2. Click here for file

Additional file 2 **Table S1: Oligonucleotide primers used for real time qRT-PCR validation of microarray results**. Click here for file
