# Supplementary material for: Genome-wide transcriptional profiling of peripheral blood leukocytes from cattle infected with Mycobacterium bovis reveals suppression of host immune genes
Source: BMC Genomics. 2011 Dec 19;12:611. doi: 10.1186/1471-2164-12-611 (PMC3292584; doi:10.1186/1471-2164-12-611)
Supplement: Additional file 1 — Perl script used for bootstrapping of cluster analysis results in Figure 2. [file 1471-2164-12-611-S1.DOC]

**Additional file 1: Perl script used for bootstrapping of cluster analysis results in Figure 2**

#! /usr/bin/perl

#Open and read datafile containing all normalised and informative probe sets

my $datafile = shift @ARGV;

open (DATA_IN,$datafile) or die "can't open file'$datafile':$!";

#Read in content line by line, first row is the header row

my $header = <DATA_IN>;

my @data = <DATA_IN>;

close DATA_IN;

my $samples = @data;

# Number of bootstrap iterations required

my $bootstrap = 1000;

#Set-up output file

my $output_file = 'outfile.txt';

my $support = 0;

foreach $cycle (1..$bootstrap) {

# generate new input and write to output file

open OUT, ">$output_file" or die "can't write to '$output_file':$!\n";

select OUT;

print "$header";

#"number of lines:".@data."\n";

#loops through data

foreach (1 ..$samples) {

my $index = rand(@data);

print $data[$index];

}

close OUT;

# generate a tree in R

# 1. create R script

my $r_script = 'making_new_expression_set_in_R.txt';

# run R on the command line

# my $job = "/cygdrive/c/Program\ Files\ \(x86\)/R/R-2.10.0/bin/Rcmd.exe BATCH CMD $r_script";

# after making symbolic link (ie shortcut) from Prog files->R->R2.10 etc to /usr/local/bin/R, use syntax below instead

my $job = "/usr/local/bin/R --no-save --args $output_file bootstrap_results.${cycle}.txt < $r_script > bootstrap.${cycle}.log";

print STDERR "$job\n";

system($job);

# read in file with tree label order

# check for support

$support++ if ('');

}

# calculate overall support
