# Supplementary material for: Genome-wide transcriptional profiling of peripheral blood leukocytes from cattle infected with Mycobacterium bovis reveals suppression of host immune genes
Source: BMC Genomics. 2011 Dec 19;12:611. doi: 10.1186/1471-2164-12-611 (PMC3292584; doi:10.1186/1471-2164-12-611)
Supplement: Additional file 2 — Table S1: Oligonucleotide primers used for real time qRT-PCR validation of microarray results. [file 1471-2164-12-611-S2.DOC]

**Additional file 2**

**Table S1: Oligonucleotide primers used for real time qRT-PCR validation of microarray results**

| **Gene Symbol** | **Forward Primer Sequence (5′-3′)** | **Reverse Primer Sequence (5′-3′)** | **Size**  **(bp)** | **RefSeq** | **Probe ID**  **(Affymetrix)** |
| --- | --- | --- | --- | --- | --- |
| *CASP1* | GAACTGCCCAGCTTTGAAGG | CCCATACCATCCCTTGCTTC | 80 | XM_002692921 | Bt.26983.1.S1_at |
| *CD83* | GGAGAAATCGACAGGCCAAG | CTTCACGGCGGGAAATTAGT | 67 | NM_001046590 | Bt.3841.1.S1_at |
| *CTLA4* | ccagagtcatgggacttggt | gaggctgggtcacattcatc | 107 | NM_174297 | Bt.19143.1.A1_at |
| *DEFB10* | cggaacctggcaccttttta | gcaggaggagcaggtaatgg | 103 | NM_001115084 | Bt.26980.1.S1_at |
| *IFNB1* | AGGAGCTACAGCTTGCTTCG | CGAGGCAATGTTGAGGAGTT | 97 | NM_174350 | BtAffx.1.7.S1_at |
| *IFNAR1* | ACCTCCTTCCTCTGTTGACG | CATCTTTCCGTTTGTTCCTCA | 87 | NM_174552 | Bt.4557.1.S1_at |
| *IFNG* | TCAAATTCCGGTGGATGATCTGC | GACCATTACGTTGATGCTCTCCG | 150 | NM_174086 | Bt.188.1.S1_at |
| *IFNGR1* | AAGCGAGAAGGCACCAAGTT | GGCAATTGGCTGGTAGGTG | 114 | NM_001035063 | Not applicable |
| *IFNGR2* | TTTCCTTGGTTCTGGCAAGG | GACAGGAGAGGCTGGGACAG | 62 | XM_592804 | Bt.4251.1.S1_at |
| *IL1A* | TTCGAGATATGTCAGGTCCATACC | AGTCACAGGAAGCTGAGAATCC | 116 | NM_174092 | Bt.191.1.S1_at |
| *IL8* | CTTTTGAGATGCCTGGCCTA | GGAAAGACAGTCTCAAGATTCACA | 79 | NM_173925 | Bt.155.1.S1_at |
| *IL15* | GGCTGGCATTCATGTCTTCA | GCCAGTTTGCTTCTGTTTTGG | 68 | NM_174090 | Bt.522.1.S1_at |
| *JAK1* | CCATCGAAACCGATAAGGAG | CCGAAAGACCAGACATCAGAG | 124 | NM_001206534 | Bt.9115.1.S1_at |
| *KIR3DS1* | AGAAGCACGGCTTCCTCAAG | ACAGCGAGGAAGATGCTGGT | 93 | NM_001008415 | Bt.29866.1.S1_at |
| *MYD88* | GGAGCCCAGGACCCTAAATC | GCCTCTGACCCTCCTGAGAC | 109 | NM_001014382 | Bt.20297.1.S1_at |
| *PTPN2* | GGAGCGAAGTTTATAAAGGGAGA | TTGGCGAATGATCAAAAACA | 94 | NM_001035431 | Bt.5590.1.S1_at |
| *STAT1* | AGAGGTGCTGAGTTGGCAGT | TTGTCCATGGAATCAGACCA | 125 | NM_001077900 | Bt.9064.1.S1_at |
| *STAT2* | TTAATCCTGAGGAGCGGAAG | GGCAGTTGCTGCAGTTCAT | 85 | XM_588270 | Bt.27943.1.S1_at |
| *TLR3* | TGGAAGCACTTCTCCCCAAT | GGACACCTGCCTCAAAGTCC | 82 | NM_001008664 | Bt.12298.1.S1_at |
| *TLR4* | cccctgatgtgcttctttcc | tcgcgtaccactgaatcacc | 109 | NM_174198 | Bt.9030.1.S1_at |
| *TREM1* | TGCTTTGTCCCCTCACAAGA | CAGGGAAGTCCCAGGAAGTG | 81 | NM_206970 | Bt.9208.1.S1_at |
| *TYK2* | TCCTTTGGGGTCCTCATGT | AGGCCTATGAGCTCGATGAA | 86 | NM_001113764 | Not applicable |
| *TYROBP* | gcgacctcaacacacagagg | cgcatgcatcaggaatgact | 94 | NM_174627 | Bt.8939.1.S1_at |
| *RPL19* | CCTGAAGGTGAAGGGTAACG | CGGGCTTCCTTGGTCTTAG | 138 | NM_001040516 | Not applicable |
| *PPIA* | CATACAGGTCCTGGCATCTTGTCC | CACGTGCTTGCCATCCAACC | 108 | NM_178320 | Not applicable |
